# The discovery of archaea: from observed anomaly to consequential restructuring of the phylogenetic tree

**DOI:** 10.1007/s40656-024-00616-8

**Published:** 2024-03-26

**Authors:** Michael Fry

**Affiliations:** Department of Biochemistry, Rappaport Faculty of Medicine, Technion–Israel Institute of Technology, Efron St., Bat Galim, POB 9649, Haifa, 31096 Israel

**Keywords:** Scientific discovery, Microbial phylogenetics, Ribosomal RNA-based phylogenetics, Archaea, Carl Woese

## Abstract

Observational and experimental discoveries of new factual entities such as objects, systems, or processes, are major contributors to some advances in the life sciences. Yet, whereas discovery of theories was extensively deliberated by philosophers of science, very little philosophical attention was paid to the discovery of factual entities. This paper examines historical and philosophical aspects of the experimental discovery by Carl Woese of archaea, prokaryotes that comprise one of the three principal domains of the phylogenetic tree. Borrowing Kuhn’s terminology, this discovery of a major biological entity was made during a ‘normal science’ project of building molecular taxonomy for prokaryotes. Unexpectedly, however, an observed anomaly instigated the discovery of archaea. Substantiation of the existence of the new archaeal entity and consequent reconstruction of the phylogenetic tree prompted replacement of a long-held model of a prokarya and eukarya bipartite tree of life by a new model of a tripartite tree comprising of bacteria, archaea, and eukarya. This paper explores the history and philosophical implications of the progression of Woese’s project from normal science to anomaly-instigated model-changing discovery. It is also shown that the consequential discoveries of RNA splicing and of ribozymes were similarly prompted by unexpected irregularities during normal science activities. It is thus submitted that some discoveries of factual biological entities are triggered by unforeseen observational or experimental anomalies.

## Scientific discovery: philosophical issues

Scientific discovery of new theories or factual entities, (objects, systems, processes, etc.,) is a component of some, but not all, multilayered endeavors of gaining new scientific knowledge about the natural world.^1^ Opinions diverge on the boundaries of the term ‘discovery’.^2^ Whereas some thinkers saw discovery as the instance of new scientific insight, (“eureka moment”) others stipulated that a discovery of new scientific theory or entity becomes recognized as such only after withstanding testing and substantiation.^3^

Whereas discovery of new theories and hypotheses was the subject of extensive philosophical discourse,^4^ philosophers paid much less attention to the philosophy of discovery of factual entities. Three factors contributed to this asymmetry. First, because philosophy of science of the nineteenth and twentieth centuries took theory-governed physics as its dominant model of scientific research, (Popper, 1959, 2002; Carnap, 1966; Kuhn, 1970b; Chalmers, 2013) discovery of theories was thought to be the primary impetus for scientific inquiry. Second, under this theory-first viewpoint versions of the theory-driven hypothetico-deductive method became heuristic norm of scientific research (Popper, 1959, 2002; Platt, 1964; Godfrey-Smith, 2003). Third, because of their vastly heterogeneous histories and intricate technical specificities, experimental discoveries of factual entities resisted systematic philosophical analyses.

This paper examines a prominent case of experimental discovery of an unforeseen major biological factual entity: archaea, a third super-kingdom (domain) of the evolutionary tree of life, (Sect. 2). This and analogous discoveries in biology, (Sect. 3) are put into philosophical context in Sects. 1 and 3.

### Discovery as part of an overall context of scientific progress

Discovery of new theories or factual entities is an important contributing element in many cases of scientific advancement (Schickore, 2022). Progress in science poses  difficult and unsettled philosophical issues, involving profound questions such as what progress is, how is it made, and what distinguishes progress from other human intellectual undertakings. These unresolved questions notwithstanding, beginning with Francis Bacon, philosophers of the modern era have framed different general models of scientific progress. Some of the more enterprising models were offered in the last 70 years, (i.e., Popper, 1959/2002, 1965; Lakatos, 1968, 1970; Feyerabend, 1970, 1993; Kuhn, 1962b; Chang, 2007, 2017). Selected cases in the histories of physics, chemistry, and biology appeared to conform to one or another model. However, because of the high divergence of the theoretical and empirical paths to epistemological justification, no single model could serve as a universal template for all cases of scientific advancement.

Kuhn’s model of the progression of science, (Kuhn, 1962b, 1970a, 1970b), distinguished between ‘normal’ and ‘revolutionary’ phases of scientific research. The normal science phase was perceived as ‘puzzle solving’ activity that takes place under an umbrella of a consensually accepted theoretical and methodological framework (a ‘paradigm’ which is distinct from a model). However, according to Kuhn, anomalies that accumulate during normal scientific activity ultimately reach a level that contravenes the accepted paradigm. At such a breakpoint, reached through accumulation of many rather than a single anomaly, a new paradigm must be framed that can successfully accommodate and explain both the old and new (‘anomalous’) information. According to Kuhn, such paradigm shift entails incommensurability, i.e., stark epistemological incompatibility between the old and the new paradigms.

Kuhn drew support for his model from selected cases from the histories of physics and astronomy. However, in time the Kuhnian original model underwent adjustments and modifications, (including Kuhn’s own) and was subjected to various criticisms [i.e., (Bird, 2002, 2005; Chike, 2021; Levit & Hossfeld, 2022; Sankey, 1993; Shan, 2020; Weinert, 2014)]. Also, the historical patterns of some landmark discoveries in the life sciences ill-fitted this framework (Fry, 2016b). Without delving into the question whether the discoveries of archaea, RNA splicing, and ribozymes fully or even partially conform to the Kuhnian framework, this paper borrows from his theory the concepts of ‘normal science activity’ and ‘anomaly’ as useful constructs for the historiographies of these three discoveries.

It is difficult, and likely impossible, to craft a philosophical model of scientific progress that can serve as a universal template for the highly variable histories of discoveries of factual biological entities. Yet, comparative examination of the histories of specific cases may reveal shared patterns of selected discoveries. This paper presents detailed history of the discovery of archaea, (Sect. 2) and outlines the histories of the initial stages of the discoveries of RNA splicing and split genes and of ribozymes, (Sect. 3). It is shown that these three landmark discoveries were similarly instigated by detection of unforeseen anomalies during normal science activity.

### Philosophical notions on discovery of new hypotheses and theories

Contrasting the seventeenth century Baconian and Newtonian ideas that theories are born out of observation and experiment, the nineteenth century thinkers John Herschel and William Whewell placed generation of new scientific hypotheses ahead of experiment [(Herschel, 1831); (Whewell, 1847, 1858); for historical perspective see (Schickore, 2006)]. Whewell also contended that the instance of discovery of new scientific hypothesis is an inimitable occurrence that cannot be formulated into algorithm (‘maxim’) for the generation of unrelated other discoveries [(Whewell, 1858), aphorism III, section IV, p. 44]:

Scientific discovery must ever depend upon some happy thought, of which we cannot trace the origin; some fortunate cast of intellect, rising above all rules. No maxim can be given which inevitably lead to discovery.

Whewell also argued that beyond the initial ‘happy thought’, a discovery must include elements of articulation, development, testing, and corroboration.

In their venture to delineate the logical boundaries of science, early and mid-twentieth century philosophers, notably those of the logical positivism school, excluded the generation of new scientific theories from the realm of rational reasoning. Rather, they considered the discovery of a novel theory to be a ‘mental jump’ [(Wisdom, 1952), p. 49], ‘free mental creation’, [(Einstein, 1954), p. 291] ‘an irrational element’, or ‘creative intuition’, [(Popper, 1959/2002) p. 8]. Novel theories were thus seen as products of creative invention and not as outcomes of observed facts [(Hempel, 1966), p. 15].

Framed by the logical empiricist Hans Reichenbach, a two contexts model of discovery and corroboration of theories had significant impact on modern thinking on the discovery of scientific theories. This model divided the process of theory formation into an initial *context of discovery* of theory creation, and a subsequent *context of justification* in which the theory is evaluated, tested, and epistemically substantiated [(Reichenbach, 1938) pp. 6–7]. Because Reichenbach considered the context of discovery to be an intuitive a-rational instance, he relinquished philosophical analysis of this phase, consigning its study to psychologists, historians, and sociologists. Also, considered unamenable to logical analysis, the context of discovery could not produce a general algorithm (a so-called “discovery machine”) for the generation of other discoveries. Reichenbach claimed that unlike the irrationality of the context of discovery, the context of justification was governed by domain-neutral rules of logic and was thus open to normative formulation [(Reichenbach, 1951), p. 231]:

The act of discovery escapes logical analysis; there are no logical rules in terms of which a "discovery machine" could be constructed that would take over the creative function of the genius. But it is not the logician's task to account for scientific discoveries; all he can do is to analyze the relation between given facts and a theory presented to him with the claim that it explains these facts. In other words, logic is concerned only with the context of justification.

The two-contexts model was subjected to many criticisms and modifications. Some philosophers disputed its sharp division between discovery and justification, arguing that the two contexts were intertwined [(Feyerabend, 1970) pp. 70–71; (Feyerabend, 1993) Chapter 4; (Mowry, 1985; Nickles, 1980; Schaffner, 1974)].^5^ Other thinkers maintained that accurate description of the process requires addition of one (Curd, 1980; Kordig, 1978; Laudan, 1980; Nickles, 1980; Schaffner, 1980) or even two (Goldman, 1983) intermediate contexts.

Some philosophers challenged the idea of a completely a-rational context of discovery, suggesting that it is guided by variants of abductive logic (Aliseda, 2006; Gabbay & Woods, 2005; Hanson, 1958a, 1958b, 1961; Magnani, 2001, 2009; Paavola, 2004, 2006). Other thinkers, however, rejected abduction as logical basis for discovery (Achinstein, 1970, 1987; Frankfurt, 1958; Harman, 1965, 1968; Kapitan, 1992; Nickles, 1980). Computational and artificial intelligence methods were offered more recently as potential generators of logical rules of discovery and of discovery-producing algorithms (Addis et al., 2016; Džeroski et al., 2007; Langley, 2000; Sozou et al., 2017).

Conversely, some philosophers argued that the context of justification is not entirely logical and that it involves unreasoned steps of raising intermediary auxiliary hypotheses and assessing their testability (Nickles, 1980, 1985; Putnam, 1991).

Despite the various criticisms and modifications, Reichenbach’s original two contexts model and variants thereof are still central to much of the philosophical thought on discovery of scientific hypotheses and theories (Hoyningen-Huene, 1987; Schickore & Steinle, 2006).

### Philosophical ideas on discovery of theories in biology

Taking cue from philosophical thinking on discoveries in physics, the relatively few philosophical studies of discovery in biology have dealt with genesis and justification of new biological theories and not of factual entities.

#### The repressor theory of negative regulation of gene expression

Based on results of their experiment on the inducible expression of the enzyme β galactosidase in *E. coli*, Arthur Pardee, François Jacob, and Jacque Monod framed a theory of repressor-controlled negative regulation of gene expression (Pardee et al., 1959).^6^ Kenneth Schaffner contended that the construction of this theory was neither consistent with the standard hypothetico-deductive model nor with the scheme of sharply differentiated contexts of discovery and of justification (Schaffner, 1974). He argued first that this theory was deduced from experimental results and was not the product of irrational ‘creative intuition’ as postulated in the hypothetico-deductive model. Second, the contexts of discovery and justification^7^ similarly entailed empirical and extra-empirical factors and inferences, making both a single continuum guided by unitary logic (Schaffner, 1974). Reexamining the historical evidence, Marcel Weber contested the thesis of a single discovery-justification continuum. In his reading the logic behind the genesis of the repressor theory differed from the reasoning of its justification. He also argued that the repressor model was generated by analogy to prior cases of enzyme repression and not by deduction from experimental results [(Weber, 2005) pp. 55–63].

#### *Proposed models of logic-driven discoveries of biological theories*

Lindley Darden delineated several potential reason-based procedures for the discovery of explanatory theories for unresolved problems in genetics and molecular biology. Under one model, inexplicable observation or data are solved by application of solutions to settled analogous past problems (Darden, 1980, 1982). It was later conjectured that inter-field connections may be better sources for new theories than solved past problems (Darden, 1980, 2006; Darden & Craver, 2002). Another reason-based model employed hindsight from historical cases to mold allegedly sufficient and general non-algorithmic strategy for the discovery of biological mechanisms^8^ (Darden, 1991, 2002, 2006, 2009). Taking the change of genetic theory from Mendel to Morgan as a test case, Darden contended that although her account did not correspond to the reasoning that geneticists of the period actually employed, it still could have generated similar historical change (Darden, 1991). Weber criticized, however, both the failure of the proposed procedure to faithfully reconstruct historical scientific developments and its claims of sufficiency and generality [(Weber, 2005), pp. 63–71].

### Kuhn’s philosophical thinking on observational and experimental discovery of factual entities

In a relatively less noticed article that coincided with the first edition of his *Structure of Scientific Revolutions*, (Kuhn, 1962b) Kuhn considered the largely neglected philosophical issue of theory-free empirical discoveries of factual entities (Kuhn, 1962a). Dissecting historical cases of unpredicted observational or experimental discoveries, Kuhn outlined their general structure. Although all the studied cases were taken from the histories of physics and chemistry, his conclusions are highly relevant to discoveries in the life sciences.

Kuhn first made a distinction between two types of discovery. One kind were discoveries of objects already predicted by theory,^9^ whereas discoveries of the other type, (which is common in biology) were not predicted by prior theory.^10^ Because they are entirely unanticipated, discoveries of the second category frequently catch the scientific community by surprise (Kuhn, 1962a). Generalizing from examined historical cases, Kuhn identified three shared features of discoveries of objects that were not predicted by theory. (a) Such discoveries begin with observational or experimental findings of anomalies. Although other scientists may also encounter such irregularities, only individuals with required aptitude and gift fully notice the anomaly and pursue its significance.^11^ (b) In a second extended phase of additional observations and experimentation, the investigator strains to turn the anomaly into an established part of nature. (c) In a third and final stage, the discovery and its broader significance are adjusted, adapted, and assimilated by the professional community. Kuhn contended that by accepting the full implications of the discovery, scientists gain a new look at what was previously known (Kuhn, 1962a).

## The discovery of archaea: puzzle-solving normal science unpredictably heralded restructuring of the phylogenetic tree

This paper contemplates the history and philosophical implications of the discovery by Carl Woese in the 1970s of archaea, a third domain of the phylogenetic tree. This discovery led in time to reconstruction of the tree from a two-branched one, comprised of prokarya and eukarya, to a tripartite tree of bacteria, archaea, and eukarya. The principal focus of this contribution is the history and philosophical connotations of the progression of Woese’s project from a problem-solving ‘normal science’ investigation to anomaly-instigated discovery.

Carl Woese, (1928–2012) studied physics and mathematics at Amherst College and completed in 1953 a PhD research thesis in biophysics at Yale University. After sojourns at the University of Rochester, Yale, and General Electric Research Laboratory in Schenectady NY, he joined in 1960 the University of Illinois at Urbana-Champaign which remained his academic home until the end of his career. There he served as professor at the Institute of Genomic Biology which was posthumously renamed in his honor in 2015 ‘The Carl R. Woese Institute of Genomic Biology’.^12^ The original aim of Woese’s work at the University of Illinois was to establish evolution-based taxonomy of prokaryotes by comparing their ribosomal RNA sequences. Although this project was marked by evolutionary approach to construction of classification and by application of innovative methodology, it was nevertheless framed by theory and practice of ‘normal’ molecular biology and microbiology of the time. However, several years into the project an unexpected anomalous finding heralded the discovery of archaea, a hitherto unknown principal domain of the evolutionary tree. Substantiation and gradual acceptance of this discovery ended in the replacement of a previous scheme of a two-domain, (prokarya-eukarya) phylogenetic tree by a new model of tripartite, (bacteria-archaea-eukarya) tree. Kuhn’s terminology of normal (puzzle solving) science and paradigm-changing scientific revolution, [(Kuhn, 1970a); (Kuhn, 1970b) pp. 23–42] suits the progression from normal science of constructing molecular taxonomy for prokaryotes to the consequential discovery of archaea. This paper contends that this and other discoveries in the life sciences conform with Kuhn’s idea of anomalies as instigators of discoveries that are unpredicted by prior theories [(Kuhn, 1962a) and 1.3].

### Woese’s initial aim: construction of bacterial phylogenetic tree

On a backdrop of disconcerting failure to establish evolutionarily meaningful bacterial taxonomy, (*2.1.2*) Woese launched in the late 1960s a research project to construct molecular-based prokaryotic phylogenetic trees (Albers et al., 2013; Gold, 2014; Goldenfeld, 2014). To put this endeavor in context, the next section briefly summarizes the history of taxonomy of all forms of life and particularly of prokaryotes.

#### Changing schemes of the tree of life

The earliest taxonomic system apportioned each living thing to one of two kingdoms—Plantae (plants) or Animalia (animals) (Whittaker, 1969). This classification was capsized in the seventeenth century when Antonie van Leeuwenhoek used his simple microscope to auspiciously discover a hitherto hidden vast world of miniscule unicellular organisms that he named animalcules. Merging this new knowledge with a large body of embryological, palaeontological, and systemic data, Ernst Haeckel constructed in the nineteenth century a new phylogenetic tree of life (Dayrat, 2003). The root of this tree represented a presumed common primordial ancestor of all living things and its trunk branched into three super-kingdoms (domains): Protista (unicellular organisms that do not form tissues), Plantae, and Animalia. Because both bacteria and blue-green algae lacked cell nucleus, Haeckel merged them into a single group, the Monera which ranked below the Protista (Haeckel, 1866). Herbert Copeland later argued that the differences between Monera and Protista warranted their separation into two distinct super-kingdoms and his proposed version of the phylogenetic tree had thus four super-kingdom branches: Monera, Protista, Plantae, and Animalia (Copeland, 1938, 1947). Robert Whittaker classified Fungi as another independent super-kingdom such that his evolutionary tree comprised of five super-kingdom: Monera, Protista, Fungi, Plantae, and Animalia (Whittaker, 1959, 1969). However, the version of the phylogenetic tree that became dominant by the second half of the twentieth century was a parsimonious scheme of mere two super-kingdom branches, Prokarya and Eukarya, terms that were originally introduced by the French biologist Édouard Chatton, (Chatton, 1938). Under his classification, prokaryotes comprised of all unicells that were devoid of nucleus. However, in his terminology, eukaryotes were only monocellular nucleus bearing protists, (protozoa).^13^ Roger Stanier later expanded the eukaryotic super-kingdom to also include multicellular plants and animals (metaphyta and metazoa). Thus, whereas under Stanier’s taxonomy the prokaryotic super-kingdom of anucleate bacteria remained unchanged, his eukaryotic domain encompassed every nucleus-containing mono- or multicellular organism (Stanier, 1961; Stanier & van Niel, 1962; Stanier et al., 1963), for historical reviews see (Katscher, 2004; Sapp, 2005). This dichotomous scheme of the tree of life was consensually adopted by biologists at the second half of the twentieth century (Corliss, 1989)*.*

#### Bacterial taxonomy appeared to be indeterminable

Following the discovery of monocellular microscopic ‘animalcules’ in the seventeenth century, it was debated for almost two centuries whether they were animals or plants. The father of modern taxonomy, Carolus Linnaeus placed them in the 1767 edition of his *Systema Naturae* under the group ‘Verms’ (worms), class ‘Chaos’ and species ‘Chaos infusorium’.^14^ Various classification systems that were introduced in the subsequent two centuries were first based on distinguishing morphologies of the microscopic organisms and, starting in the mid- to late nineteenth century, on some of their characteristic metabolic properties and distinctive biochemical constituents of different bacteria.”

The Danish naturalist Otto Friedrich Müller arranged the animalcules, (named by then ‘infusoria’) into genera and species (Müller, 1773). Believing that the infusoria comprised of diverse fixed animal species,^15^ the German zoologist Christian Gottfried Ehrenberg classified them by shape into 22 families (Ehrebnberg, 1838). Others who also adhered to the idea that infosuria comprised of fixed animal species later proposed simpler classification systems (Dujardin, 1841; Perty, 1852). Botanists of the nineteenth century rejected the perception of bacteria as animals arguing instead that they were microscopic plants akin to fungi and algae. The German botanist Ferdinand Cohn classified bacteria by shape into four main groups: Sphaerobacteria (spherical cells, later named cocci), Bacterium (rod-shaped), Desmobacteria (filament-like), and Spirobacteria (screw-shaped, later termed spirilla) (Cohn, 1872, 1875, 1876). Other investigators proposed alternative bacterial classification systems that were also based on cell morphology and metabolism. However, failure to reach agreed biological definition of bacteria^16^ and to establish evolutionarily meaningful bacterial taxonomy, (Breed et al., 1957; Buchanan, 1925; Migula, 1897, 1907; Niel, 1955; Stanier & Niel, 1941) left leading mid-twentieth century microbiologists with a sense that construction of bacterial phylogenetic tree was beyond reach.^17^

Confronting this impasse, Woese undertook to establish genealogical tree of bacteria based on evolutionary changes in the nucleotide sequence of their ribosomal RNA (rRNA). Because bacteria were evolutionarily much more ancient than eukaryotes,^18^ the project was also motivated by hope of identifying a common root of the bacterial lineage. This could in turn shed light on the nature of the last universal common ancestor (LUCA) or last universal ancestor (LUA) which Woese named the ‘progenote’ (Woese, 1970; Woese & Fox, 1977a).

#### Origins of Woese’s project

At an early phase of his work as independent researcher, Woese attempted to tackle the problem of the evolution of the genetic code. Briefly, he asked how recognition had developed during the earliest stages of life on earth between specific amino acids and their corresponding transfer RNA (tRNA) carriers (Woese, 1967). Although this endeavor proved to be unproductive, its underlining idea of evolution at the molecular level led Woese to the notion that the evolution of bacteria could be clocked by changes in component(s) of their universal and evolutionarily conserved protein biosynthesis machinery. This general idea was the basis for his ensuing successful effort to construct evolution-based taxonomy of bacteria that were then thought to belong to a single prokaryal domain. The project had at its onset both a formerly framed theoretical basis, (Zuckerkandl & Pauling, 1965a, 1965b) and an effective RNA sequencing methodology previously developed by Fred Sanger (Sanger et al., 1965). Specifically, relative evolutionary distances between different bacterial species and phyla were derived from quantified changes in the nucleotide sequences of their rRNA molecules. Graphically positioned according to their relative evolutionary distances, bacterial taxa appeared as branches of a constructed phylogenetic tree.

#### The theoretical basis to Woese’s bacterial phylogenetic project

The French biologist Emile Zuckerkandl working in the first half of the 1960s in Linus Pauling’s Caltech laboratory, catalogued substituted amino acids in homologous globin chains from different species.^19^ Positing that any particular protein evolves over time at a fairly constant rate, Zuckerkandl and Pauling hypothesized that the number of substituted amino acid in homologous proteins from two different species was proportional to the evolutionary time that elapsed since their divergence from a last common ancestor [(Zuckerkandl & Pauling, 1965a, 1965b), for history of their work and hypothesis see (Morgan, 1998)].^20^ Under this so-called ‘molecular clock hypothesis’, comparative sequence data were used to build phylogenetic trees whose taxon-representing branches were placed at evolutionary temporal distances from one another. Woese applied a modified form of this hypothesis as the theoretical basis for his construction of bacterial genealogical trees. Reasoning that proteins are not universally distributed and that they do not necessarily preserve constant function throughout evolution, Woese compared instead changes in highly conserved nucleotide sequences of 16S rRNA molecules of different bacterial species. This RNA was considered a more reliable evolutionary chronometer because: (a) a clocklike behavior was guaranteed by the nearly random nature of changes in its sequence. (b) Sequence changes in paired species were proportional to their evolutionary distances. (c) The size of rRNA was large enough to yield quantitively dependable information.

#### Sanger’s RNA sequencing technique provided Woese with a vital experimental tool

To determine nucleotide sequences of bacterial rRNA, Woese and associates adopted a technique for the sequencing of short RNA fragments, (Sanger et al., 1965). Employing this method, they compared partial sequences of rRNA molecules from different species of bacteria. Extents of rRNA sequence variance between paired bacterial species were used to calculate their relative evolutionary distances in constructed phylogenetic trees (*2.1.7*).

**Fig. 1:**
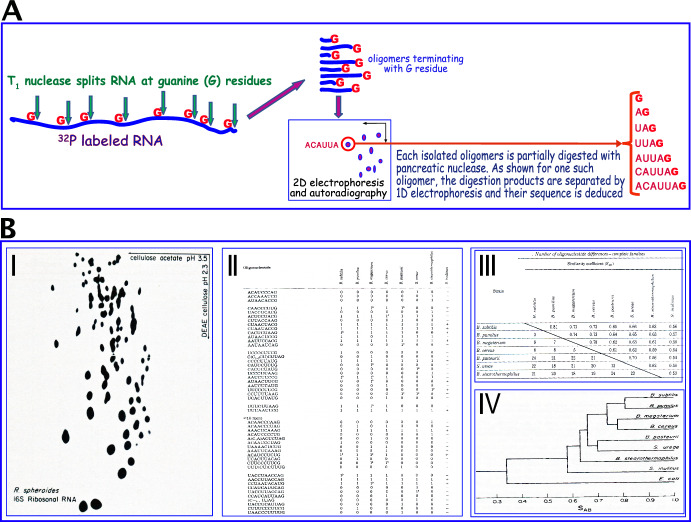
Stepwise construction of dendrogram of evolutionary distances between different species of bacteria. **A** Scheme of the 16S rRNA sequencing technique (see *2.1.5* for details). **B** Steps in building dendrogram of evolutionary distances. **B (I)** Typical pattern of two-dimensional electrophoretic separation of 16S rRNA-derived oligonucleotides [from (Zablen & Woese, 1975)]. **B(II)** Partial catalogue of 16S rRNA oligonucleotides of eight different bacterial species. **B (III)** Matrix of similarity coefficients of 16S RNA sequences from the different bacterial species. **B (IV)** Dendrogram of evolutionary distances between the bacterial species as gauged by their similarity coefficients. Figures **B (II)** to **B (IV)** were adapted from (Fox et al., 1977b)

Figures 1A and 1B (I) illustrate main elements of Woese’s methodology. Briefly, to label their RNA, bacteria were fed a radioactive ^32^P isotope of phosphorous. Next, a desired ^32^P-labeled RNA species, (i.e., 5S rRNA, 16S rRNA etc.,) was isolated and then digested by T1 ribonuclease (RNase) that specifically nicked it terminally to each of its randomly situated guanine (G) residues (Fig. 1A). Oligomeric products of the enzymatic digestion were placed on paper and separated according to their lengths, base composition, and sequences by electrophoresis in two dimensions. Viewed by autoradiography, differently migrating spots of radioactive RNA oligomers formed typical ‘fingerprint’ patterns such as shown in Fig. 1B(I). In a final step, each spot was cut out, partially digested by ribonucleases other than T1 and its nucleotide sequence was determined by one dimensional electrophoresis of the digestion products (Fig. 1A).

#### Woese first presented his core ideas in a 1969 letter to Crick

Because of their shared interest in the evolution of the genetic code, Crick and Woese corresponded on occasion in the 1960s and 1970s.^21^ Pertinent to this study is a 1969 letter^22^ in which Woese presented his thoughts on mapping evolutionary distances between bacterial species based on sequence changes in their genes:

If we ever to unravel the course of events leading to the evolution (i.e., simplest) cells, I feel it will be necessary to extend our knowledge of evolution backward in time by billion years or so – i.e., backward into the period of actual “Cellular Evolution”. There is a possibility, though not a certainty, that this can be done by using the cell’s “internal fossil record” – i.e., the primary structure of various genes.

Woese further speculated that evolutionary change is best reflected by changes in sequences of RNA component(s) of the translation machinery:

The obvious choice of molecules here lies in the components of the translation apparatus. What more ancient lineages are there? A priori it seems impossible to evolve any structural gene without the capacity to translate the gene - making the evolution of some rudimentary translation machine necessarily a very early happening. Hopefully that machine was a direct lineal ancestor (both functionally and structurally) of the present one. Also, I feel (and you may too) that the RNA components of the machine hold more promise than (most of the) protein components.

While writing this letter Woese had already decided to use Sanger’s method for the sequencing of bacterial RNA.^23^ However, being aware of his lack of the required technical expertise, he asked for Crick’s help in recruiting: “…some energetic young product of Fred Sanger’s lab,^24^ whose scientific capacities complement mine”. Eventually, however, no such a person was required as his group became proficient in the use and even the improvement of Sanger’s short RNA sequencing technique.

#### Variance in rRNA sequences was used as molecular clock to build bacterial phylogenetic trees

Starting in the early 1970s, Woese and associates embarked on yearslong experimental efforts to build genealogical trees for bacteria. Phyla and species were respectively represented as branches and sub-branches of the tree. The branches and sub-branches were placed according to their relative evolutionary distances that were proportional to the degrees of disparity between their rRNA sequences. Since ribosomes are components of the universal and indispensable translation apparatus, their RNA constituents were thought to have changed during evolution at more restrictive and slower rate than any other gene/protein.^25^ It was thus reasoned that extents of change in nucleotide sequences of rRNA of different bacteria best reflect elapsed evolutionary time. Additionally, it was theoretically possible that monitoring rRNA sequences of diverse species could uncover evolutionarily early molecules (‘molecular fossils’) that may potentially unveil attributes of ancient versions of the translation machinery.

Because of the relative ease of the isolation, characterization, and sequencing of shorter RNA molecules, Woese initially compared in different bacterial species sequences of the ~ 120 nucleotide-long molecules of 5S ribosomal RNA^26^ (Sogin et al., 1972). It was soon realized, however, that these molecules were too short to yield large enough numbers of mutational changes. He therefore switched to comparisons of sequences in different bacteria of the ~ 1500 nucleotides-long 16S RNA component of the smaller ribosomal subunit.^27^ Because this RNA species exists in every organism and its function is conserved, it was deemed ideal evolutionary ‘chronometer’ that reflects true line of descent. This premise could potentially be wrong if horizontal (interspecies) gene transfer was a major contributor to genetic variation. However, evidence indicated that horizontal transfer of genes had negligible effect on the accuracy of evolutionary clocks based on 16S rRNA, (Woese et al., 1980a) or cytochrome C, (Dickerson, 1980).

In carrying out this project Woese and associates^28^ had to overcome numerous technical hurdles. First, the laboratory had to learn the Sanger RNA sequencing technique. This was done when David Bishop, past trainee of Sanger’s and then postdoctoral fellow in the neighboring Spiegelman laboratory, taught the technique to Mitchell Sogin, a graduate student in the Woese laboratory (Pace et al., 2012). Then there was the problem of obtaining different bacterial species. Because he was not a microbiologist himself, Woese forged contacts with bacteriologists in other institutions who provided him with different species of bacteria. The various bacterial strains were aerobic or anaerobic, prototrophic, or heterotrophic, with each type having its individual nutritional requirements. It was thus technically nontrivial to establish conditions for cell growth and division at rates that allowed incorporation of high enough levels of ^32^P into their rRNA. Once grown and properly labeled, cells were disintegrated and their radioactive 16S rRNA molecules were isolated. Following enzymatic digestion of the rRNA, product oligonucleotides were separated by 2D electrophoresis, viewed by autoradiography, isolated, and their nucleotide sequences were determined (Figs. 1A, B (I)). Typical comparison of a set of 16S rRNA oligomeric sequences of eight different species is shown in Figs. 1B (II-IV). First, frequencies of occurrence of identical fragment sequences were tabulated, (Figs. 1B (II)). Next, binary association coefficients, S_*ab*_, were calculated and charted (Fig. 1B (III)).^29^ Finally, rRNA sequences of the different species were graphically arranged by their S_*ab*_ values in a dendrogram of relative evolutionary distances between species (Fig. 1B (IV)).

For more than a decade, Woese and his coworkers tenaciously applied the described multi-step procedure for numerous bacterial species.^30^ Years later Woese described his tedious routine (Woese, 2007):

My job was to determine the complete sequence of every oligonucleotide of significant length (five or more nucleotides) in primary pattern,^31^ which required the aforementioned “secondary” patterns. These in turn were created by removing little snippets of paper in the appropriate places in the original electropherogram and further digesting the oligonucleotide(s) therein (*in situ*) with one or a few ribonucleases of different cutting specificities than that of T1 RNase […] From the one or several “secondary” taken from a primary spot, the exact sequence of the oligonucleotide(s) in the corresponding primary spot could (almost always) be deduced [….]

“Reading” a Sanger pattern was painstaking work, requiring a good fraction of the day to work up a single primary,” something I at the time had been doing for several days a week off and on for a long time. It was routine work, boring, but demanding full concentration. (There were days when I would walk home from work saying to myself: “Woese, you have destroyed your mind again today”).

The project was modestly opened in 1972 by comparison of catalogues of sequences of oligomeric products of T1 RNase digestion of 16S rRNA from *Escherichia coli* and *Bacillus megaterium* (Pechman & Woese, 1972). Soon thereafter Woese and associates modified and improved some aspects of the RNA sequencing method, (Uchida et al., 1974; Woese et al., 1976) and the scope of analyzed bacterial species was gradually expanded. Sequences of 16S rRNA were determined for diverse bacterial families that included, among others, Enterobacteriaceae, (Woese et al., 1974; Zablen et al., 1975) Cyanophyta, (Blue-Green algae), (Doolittle et al., 1975) photosynthetic bacteria, (Zablen & Woese, 1975) mesophilic and thermophilic Bacillaceae, (Fox et al., 1977b; Woese et al., 1976) and Mycoplasma (Woese et al., 1980b).^32^ By 1975 the Woese group had sequenced 16S rRNA oligonucleotides from about 30 bacterial species and by the end of that decade the number grew to 100 species (Woese, 2013).^33^ Calculated association coefficients placed different bacterial species at relative evolutionary distances from one another, allowing construction of genealogical trees.^34^ 16S rRNA-based bacterial systematics divide the eubacterial world into ten divisions, (phyla) each with its own subdivisions (Woese, 1987; Woese et al., 1985).

#### What did the phylogeny project achieve and what remained unsolved

At the time that Woese launched his phylogeny project in the late 1960s microbiologists had abandoned hope of establishing methodical taxonomy of bacteria. Instead, practitioners of medical, agricultural, and industrial bacteriology unsystematically grouped bacteria by their morphology and metabolism (*2.1.2*). Woese held, however, that rational bacterial classification system must be informed by evolution. Thus, taxa and individual species should be arranged in a phylogenetic tree according to their relative evolutionary distances. He further reasoned that such distances are best clocked by monitoring inter-species changes in the 16S rRNA elements of the universal and conserved translation machinery (*2.1.7*). It soon became evident that 16S rRNA-based taxonomies conflicted with phenotype-based phylogenetics. Thus, 16S RNA systematics identified some phenotypically similar bacteria as members of different phyla. Conversely, 16S rRNA genealogy categorized some phenotypically dissimilar species under the same divisions and subdivisions. Later replacement of the Sanger/Woese RNA sequencing method by newer techniques of sequencing full rRNA genes, (Brosius et al., 1978; Carbon et al., 1978), greatly accelerated the accumulation of 16S rRNA sequences and allowed growth of more detailed phylogenetic trees. Despite early resistance to the 16S rRNA-based phylogeny, it was gradually accepted as the standard method for estimating evolutionary distances and as the most reliable basis for building phylogenetic trees. This acceptance was highlighted in 2001 when Bergey’s Manual of Systematic Bacteriology, the benchmark of bacterial classification, changed its phenotype-based systematics to 16S rRNA-based one (Garrity et al., 2001).^35^ In a broader context, Woese’s powerful molecular approach for tracing the evolution of cells and their organelles invigorated the field of evolutionary biology at large.

A second objective of Woese’s molecular phylogeny project remained, however, unfulfilled. It was anticipated that tracing of the root of the evolutionary tree would expose the origin of all living cells, an entity that Woese and Fox named the progenote (Woese & Fox, 1977a).^36^ Already in his 1969 letter to Crick^18^ Woese insinuated that tracking bacterial evolution back to its beginning may uncover a primordial living form at the cusp of cellular evolution:

If we are ever to unravel the course of events leading to the evolution of the procaryotic (i.e., simplest) cells, I feel it will be necessary to extend our knowledge of evolution backward in time by a billion years or so -- i.e., backward into the period of actual “Cellular Evolution.

In subsequent years Woese, (Woese, 1998b, 2000, 2002) and many other researchers conjectured extensively on the possible nature of the progenote. However, to this day no common root has been identified for the bacterial, archaeal, and eukaryotic phylogenetic trees, and the nature of the progenote remains elusive.^37^

### The discovery of archaea: an unforeseen model-changing outcome of the bacterial phylogeny project

Archaea, a principal third branch of the phylogenetic tree, were unexpectedly discovered in 1976/77 during Woese’s bacterial molecular taxonomy project. This section describes this discovery, the controversy that it raised, and early phases of its substantiation and acceptance up to the early 1990s.

#### The start: experimenting with methanogenic prokaryotes

Construction of an all-inclusive phylogenetic tree necessitated a broad base of 16S rRNA sequences from diverse bacterial species. Provided with different strains of bacteria by various bacteriologists, the Woese laboratory amassed by 1976 sequences of 16S rRNA from about 60 bacterial species and from few eukaryotes.^38^ One bacteriologist with whom Woese had conferred was Ralph Wolfe, his departmental colleague and expert on methanogenic bacteria. These morphologically heterogenous and strictly anaerobic prokaryotes metabolically produce methane by reducing carbon dioxide in the presence of hydrogen. Although Woese was keen to add 16S RNA of methanogens to his catalogue, their standard culturing conditions precluded presence of high enough levels of the ^32^P isotope. George Fox, then a postdoctoral associate in the Woese laboratory, discussed the problem with William (Bill) Balch, a doctoral student in the Wolfe laboratory.^39^ Ultimately, Balch devised a method to grow methanogens anaerobically in pressurized atmosphere of carbon dioxide and hydrogen (Balch & Wolfe, 1976). Importantly, these conditions allowed addition of the necessary levels of ^32^P without exposure to oxygen or contamination (Fox, 2015; Sapp & Fox, 2013; Wolfe, 2014).

#### An anomaly set the stage for a breakthrough discovery: sequences of methanogenic 16S rRNA oligomers differed from those of known prokaryotes

A moment of revelation occurred in June 1976 when Woese had first looked at a primary fingerprint pattern of oligonucleotides that Linda Magrum and George Fox derived from 16S rRNA of a methanogenic strain. Absent from this pattern were two spots of modified oligonucleotides that hallmarked every hitherto analyzed prokaryotic 16S rRNA. Alerted by this anomalous pattern, Woese went on to determine nucleotide sequences of the resolved methanogenic rRNA oligomers. Strikingly, sequences of some oligomers were unlike those of known prokaryotes, and several were typical to eukaryotes. Here is his retrospective description of the anomaly and his sense of a significant discovery [(Woese, 2007), bold in the origin]:

The more oligos I sequenced, the less prokaryotic it felt, as signature oligo failed to turn up. However, a number of them were still there, as, surprisingly, were some oligos from the eukaryotic signature. […] I rushed to share my out-of-biology experience with George, a skeptical George Fox to be sure.^40^ […] whatever skepticism he initially evinced quickly dissipated. Yes, he agreed, there probably was something else out there: it wasn’t just prokaryotes and eukaryotes all the way down. That was a heady thought, novel enough that we sensed trouble in trying to convince other biologists of that idea. Little did we know how **much** trouble there would be.

Ralph Wolfe also described in his own words the surprise and wonder that the unanticipated results evoked (Wolfe, 2014):

…his [*Woese’s*] response was “Wolfe, these methanogens are not bacteria.” “Of course they are Carl; they look like bacteria.” “They are not related to any bacteria I’ve seen.”

Because Woese had spent 10 years developing the method and analyzing the 2-D chromatographic patterns of T_1_ endonuclease digestion patterns of ^32^P-labeled 16S rRNA from 60 different bacteria, he was easily able to discern that the methanogens were different!

Although evidence was rather slim at that early stage, Woese nonetheless was quick to proclaim that a new form of life has been discovered.^41^ The ciliatologist David Nanney and Linda Magrum of the Woese laboratory independently suggested to name these microorganisms ‘archaebacteria’. This early name was later changed to ‘archaea’, a term that this paper henceforth uses. To underscore the distinction between archaebacteria (archaea) and non-archaeal prokaryotes, the latter were christened eubacteria.

#### The discovery of archaebacteria was made public

Woese and Fox presented their first molecular evidence for the distinctiveness of archaea from eubacteria and eukaryotes in a short paper in the November 1, 1977 issue of the *Proceedings of the National Academy of Science USA* (Woese & Fox, 1977b). The evidential centerpiece of this report was a table of measured values of association coefficients (*S*_*ab*_) of paired 18S rRNA sequences from three eukaryotic species; 16S rRNA of five eubacteria; chloroplasts rRNA derived from *Lemna* aquatic plant;^42^ and 16S rRNA of four methanogenic archaeal species. These data clearly indicated that degrees of sequence similarity were significantly higher for intra-group paired sequences (i.e., eukaryotic with eukaryotic species, etc.,) than for inter-group pairs (i.e., eukaryotic with eubacterial species, etc.,). Most importantly, whereas 16S rRNA sequences of the four archaeal species had high degree of similarity, their resemblance to eubacterial or eukaryotic rRNA sequences was much lower. Based on these findings, Woese and Fox boldly proposed that the then prevailing prokaryotes/eukaryotes bipartite tree of life should be replaced by a tripartite tree that branched into ‘three aboriginal lines of descent’:^43^ (a) Eubacteria that comprised of all ‘typical’ bacteria; (b) Archaebacteria (which at that early stage comprised of only methanogenic bacteria)^44^ and; (c) Eukaryotes (Eukarya) (Woese & Fox, 1977b).^45^

#### The claim that archaea constitute a new phylogenetic domain met with skepticism and hostility

**Fig. 2:**
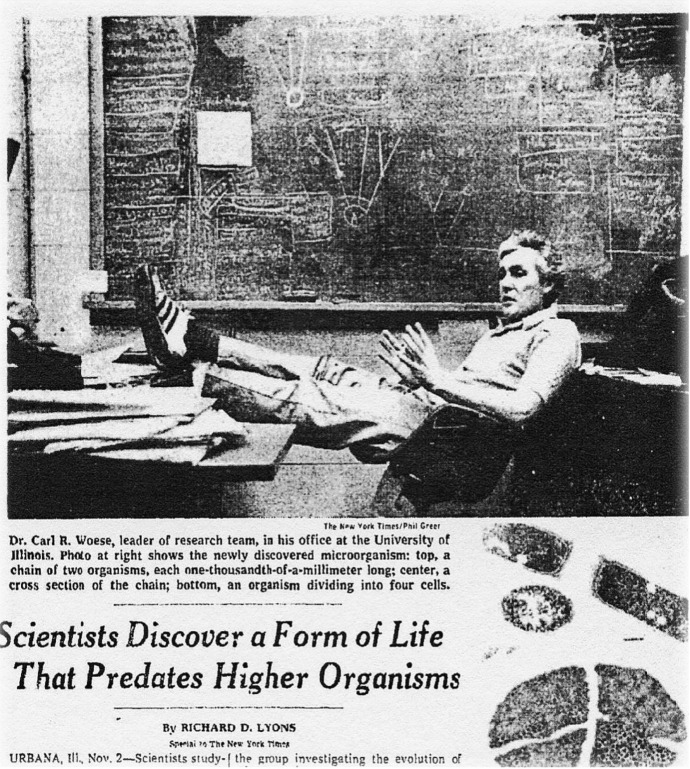
Headline of the November 3, 1977, *New York Times* front page news story on the discovery of archaea

Concurrently with the publication of the formal technical report, (Woese & Fox, 1977b) the *New York Times* published a front page news story under the heading: “*Scientists Discover a Form of Life that Predates Higher Organisms*”^46^ (Fig. 2). This audaciously headlined article prompted many microbiologists, including the 1969 Nobel Prize laureate Salvador Luria, to call and admonish Ralph Wolfe (Wolfe, 2014):

The immediate response of the scientific community to the press release was negative with disbelief and much hostility, especially among microbiologists. Scientists were suspicious of scientific publication in newspapers, and only a very few were familiar with the use of 16S rRNA oligonucleotides to define relationships among organisms. Among the phone calls that I received the morning of November 3, the one by S. E. Luria was the most civil and free of four-letter words. Luria was a Professor of Microbiology, when I joined the Department at Illinois in 1953 and had later moved to MIT.

Luria: “Ralph, you must dissociate yourself from this nonsense, or you’re going to ruin your career!”

“But, Lu, the data are solid and support the conclusions: they are in the current issue of PNAS.”

Luria: “Oh yes, my issue just arrived.”

“If you would like to discuss the paper after you have had a chance to look at it, give me a ring.”

He did not call again. I wanted to crawl under something and hide.

Secondary sources suggested that many microbiologists, who have also mostly relied on the *Times* story and not on the *Proceedings* paper, shared Luria’s negative impressions of the discovery (Morell, 1997). In general, Woese was regarded by mainstream researchers of the time as a marginal individual doing dubious science.^47^

**Fig. 3:**
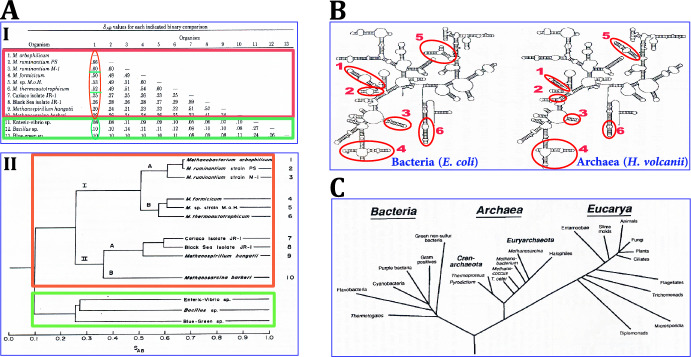
16S rRNA sequences support a model of archaea as one of three branching domains of the phylogenetic tree. **A** Construction of phylogenetic tree of archaea and eubacteria. **A (I)** Ten methanogenic archaeal species were separated by the association coefficients (S_*ab*_) of their paired 16S RNA sequences^26^ into three internally related groups of close, intermediate, and low similarity to reference sequence of *M. arbophilicum*. Yet, the 16S rRNA sequences of all three methanogenic subgroups were more similar to one another than to 16S rRNA of three eubacterial species. **A (II)** Arrangement of the eubacterial and archaeal 16S rRNA sequences by their association coefficient-derived evolutionary distances yielded a phylogenetic tree with two separate branches of archaeal and eubacterial domains [adapted from (Fox et al., 1977a)]. **B** 16S rRNA chains of a bacterium (*E. coli*) and halophilic *archaeaon* (*H. volcanii*) had different patterns of secondary structure regions modeled after (Woese et al., 1983). Locales of divergent secondary structure are marked by numbered red ellipses [adapted from (Gutell et al., 1985)]. **C** Early version of the three-domain model of the phylogenetic tree (Woese, 1993). The archaeal and eukaryotic domains were closer to one another than to the eubacterial domain (*2.2.7)*. As additional archaeal phyla were discovered, the archaeal domain in more recent versions of the tree has greater number of sub-branches (Eme et al., 2017; Hug et al., 2016)

Soon after the initial description of the four methanogenic archaeal species, (Woese & Fox, 1977b) the Woese and Wolfe laboratories collaborated to construct a phylogenetic tree from 16S rRNA sequences of 10 methanogenic archaeal species and 3 eubacterial reference species (Fox et al., 1977a). Despite being phenotypically heterogenous, all ten archaea proved to be members of sub-branching single line of descent, distinctly different from the eubacterial one (Fig. 3A). Regardless of these and subsequent corroborative results, for the next several years microbiologists and evolutionary biologists continued to contest Woese’s three-domain phylogenetic tree [for description of Woese’s struggles with largely behind-the-back criticisms see (Morell, 1997)]. Yet, the weight of progressively accumulating evidence gradually persuaded most interested scientists to accept archaea as an independent line of descent separate from the eubacterial and eukaryotic phylogenetic domains (2.2.5). Even so, some prominent evolutionists persisted in questioning the validity of rRNA-based classification and held on to the model of bipartite (prokaryotic and eukaryotic) evolutionary tree (2.2.6).

#### Amassed evidence buttressed the perception of archaea as a distinct phylogenetic domain

After his and Fox’s first report of archaea, (Woese & Fox, 1977b) Woese endeavored to experimentally establish this group as a domain separate from Eubacteria and Eukarya^48^. Indeed, he and others built up a body of experimental data that significantly strengthened this premise. The notion of archaea as distinctive evolutionary domain was independently advanced by discoveries of German and American laboratories^49^ of their idiosyncratic biochemical features. Thus, even before they have been defined as archaea, cell walls of some methanogenic, halophilic, and thermoacidophilic prokaryotes,^50^ were found to have exclusive components, (Kandler & Hippe, 1977; Kandler & König, 1978). Also, these microorganisms had distinguishing ether-linked membrane lipids, (Kates et al., 1966; Langworthy et al., 1972; Tornabene & Langworthy, 1979) eukaryotic-like components of RNA polymerase, (Zillig et al., 1978, 1979, 1980) and non-bacterial translation elongation factor (Kessel & Klink, 1980, 1982). In parallel, the Woese team showed that the 16S rRNA sequence of the halophile *Halobacterium halobium* marked it as a member of the archaeal group (Woese et al., 1978). Integrating evidence on the emblematic sequences of archaeal 5S and 16S rRNA and on the characteristic chemistry of their cell walls and lipids, Woese and associates proposed that in addition to methanogens the archaeal domain also included halophiles and thermoacidophiles (Woese et al., 1978).^51^ Subsequently gathered experimental data amply corroborated this portending proposal. One early example of such supporting evidence is shown in Fig. 3B. Here the distinction between a eubacterial species, (*E. coli*) and halophilic archaeal species, (*H. volcanii*) was demonstrated by the different folding of their 16S rRNA into secondary structures (Gutell et al., 1985).

#### Relationship of archaea to the eubacterial and eukaryotic domains

Early studies indicated that despite their prokaryotic phenotypes, archaea were more similar in some respects to eukaryotes than to eubacteria. Archaeal eukaryotic-like genes included, among others, 5S rRNA, (Hori & Osawa, 1979) subunit structure of RNA polymerase, (Berghofer et al., 1988; Huet et al., 1983) translation elongation factors, (Iwabe et al., 1989; Lechner & Böck, 1987; Lechner et al., 1988, 1989) subunits of ATPase (Iwabe et al., 1989) and some ribosomal proteins (Matheson et al., 1980). Indeed, the first completely sequenced genome of an archaeon, *Methanococcus jannaschii*, revealed that genes involved in transcription, translation, and DNA replication were comparable to their eukaryotic paralogues whereas genes related to metabolism, energy generation, and cell division, resembled those of bacteria. Importantly, however, analysis showed that the evolutionary lineage of this archaeon was different from those of eubacteria or eukarya (Bult et al., 1996). Computer aided genomic analysis of two different methanogenic archaeal species identified genes that were more similar to bacterial than to eukaryotic paralogues, others that were closer to eukaryotic than to bacterial genes, whereas a portion of the genome was exclusively archaeal (Koonin et al., 1997; Smith et al., 1997).^52^ This last group of archaea-specific protein-encoding genes constituted an archaeal genomic signature (Graham et al., 2000).

As noted, Woese’s discovery of archaea and his assertion that archaea constituted an evolutionary domain independent from the eubacterial and eukaryotic domains was initially met with skepticism and resistance. However, molecular and biochemical data gathered in the late 1980s convinced most scientists that archaea did indeed constitute a third evolutionary domain distinct from both the eubacterial and eukaryotic domains. Ultimately, a ‘three-legged stool’ model of a tripartite phylogenetic tree was adopted (Fig. 3C).^53^ For a time, it remained unknown whether the three domains were equally separated from one another or alternatively, two of the domains were closer to one another than to the third one. To answer this question, two teams applied paralogous rooting phylogenetic bioinformatics technique, (Schwartz & Dayhoff, 1978) showing that archaea and eukarya were evolutionary closer to one another than to eubacteria, (Gogarten et al., 1989; Iwabe et al., 1989) (Fig. 3C).

#### Beyond the initial discovery—current state of archaeal research

As more than 99% of all microorganisms cannot be cultivated by standard methods, (Amann et al., 1995) studies of archaea were initially hindered by inability to grow in culture most of these microorganisms. This obstacle was removed with the introduction of cloning and PCR amplification techniques that allowed isolation and sequencing of 16S rRNA genes from DNA of unculturable archaea directly collected from the environment (Amann et al., 1995; Pace et al., 1985, 1986). Detecting in this way large numbers of new types of archaea expanded the archaeal domain from just two phyla in the early 1990s, to more than 20 phyla today (Geesink & Ettema, 2022). Of the many new inroads that these advances opened, perhaps the most noteworthy was the identification of the archaeal Asgard phylum that comprises of some members that appear to be closest to the prokarya-eukarya boundary.^54^

## Normal science activities engendered other unanticipated model-changing discoveries

The discovery of archaea emerged unexpectedly during Woese’s methodological effort to construct a molecular-based evolutionary tree for bacteria. This taxonomic endeavor was framed by a model of a single prokaryotic domain that comprised of all unicells with no nucleus. This domain allegedly bifurcated in time into two-part evolutionary tree of prokaryotic and eukaryotic domains. Thus, because it operated within the consensually accepted theoretical framework of the day, the bacterial classification enterprise can be characterized as puzzle-solving normal science activity [(Kuhn, 1970a); (Kuhn, 1970b) pp. 23–42]. However, an anomaly noticed during this ‘normal science’ project, engendered the unforeseen landmark discovery of archaea. That archaea embodied a distinct domain of the phylogenetic tree was corroborated within a relatively short time. Comparative studies of molecular and biochemical features of archaea relative to those of eubacteria and eukarya, led to realization that the earliest branching point of the evolutionary tree was at the split between the archaeal and eubacterial domains. It was further recognized that this separation was followed by splitting of the eukaryotic from the archaeal domain. Thus, the traditional old view of a bipartite tree in which eukarya branched out from their prokaryotic forebears, had to be replaced by tripartite tree that first branched into eubacterial and archaeal domains and then had the eukarya split from their archaea antecedents. The extension of the tree to three instead of two domains and the new understanding of the lineages and temporal relationships between these domains forced a change from a model of a bipartite tree to a new model of a tripartite tree.

The described pattern of progression from normal science activity to emerging anomaly and then to a model-changing discovery, is not exclusive to the case of archaea. Two historical cases that are briefly described below illustrate that a similar pattern characterized the discovery of other major biological entities.

### First case: anomaly led to the discovery of split genes and RNA splicing

Systematic experimental studies in the 1960s established that molecules of phage and bacterial messenger RNA (mRNA) were colinear with their encoding genes (Sarabhai et al., 1964; Yanofsky et al., 1964, 1967). Eukaryotic cells and their viruses posed, however, a mystery since their pre-mRNA nuclear transcripts, (so-called heterogenous nuclear RNA; hnRNA) were larger by up to tenfold than the cytoplasmic mRNA molecules (Hiatt, 1962; Scherrer & Darnell, 1962). Experimental studies of the molecular structure of hnRNA and of cytoplasmic mRNA demonstrated their respective precursor-product relationships but did not answer the riddle of their different sizes [reviewed in (Fry, 2016a) pp.495–505]. The two independent respective research groups of Phillip Sharp and Richard Roberts,^55^ concomitantly solved this enigma by showing that genes of human adenovirus, (and later of eukaryotic cells) included transcribed coding segments (‘exons”) and non-coding intervening sequences (‘introns’). Following transcription of a complete gene, the hnRNA transcripts were reduced in length by removal of their introns whereas the exons were rejoined to form shorter translatable cytoplasmic mRNA molecules, [(Berget et al., 1977; Chow et al., 1977); reviewed in (Fry, 2016a) pp. 505–517]. Notably, the original aims of the Sharp and the Roberts teams were not to solve the puzzle of the different sizes of the precursor hnRNA and its mRNA product. Taking the Sharp case as an example, his original ‘normal science’ project, which was based on accepted theory and methodologies of the time, was aimed at determining which adenovirus genes were transcribed into their mRNA molecules at different stages of the virus lytic cycle. Without going into technical details, suffice it to say that Sharp and his associates used electron microscopy to view hybrids of a specific viral gene with its mRNA transcript. An anomaly was observed, however, when the electron micrographs revealed looping out of single strands of the DNA from their double-stranded hybrid regions with the mRNA (Berget et al., 1977). Arnold Berk, who was at the time a postdoctoral fellow in the lab, later described Sharp’s reaction to this unexpected ‘anomaly’ (Berk, 2016):

Sue Berget, and Claire Moore went down to the EM in the MIT Cancer Center a couple of floors below the Sharp laboratory. I was working on my own projects that day and anxiously awaited news of the results. After a couple of hours or so, Phil came back into the laboratory looking somewhat stunned, a very unusual expression for Phil. “Did you see a loop?,” I asked him anxiously. By this time I had been in the Sharp laboratory for about 9 mo, and I had never heard Phil use anything but the mildest forms of profanity. Nonetheless, Phil excitedly responded: *There are three [profanity] loops!*

Substantiation of this ‘eureka moment’-style observation, together with the independent parallel discoveries by the Roberts team, ended up in dismissal of the concept of universal colinearity of mRNA molecules with their encoding genes and in its replacement by a new model under which colinearity marked only bacterial mRNA whereas mRNA of eukarya was non-colinear.

Although different in technical details, Roberts’ project was also not originally aimed at solving the hnRNA-mRNA relationships puzzle. There too, an unexpected observed ‘anomaly’ led to an independent discovery of split genes and RNA splicing (Chow et al., 1977).

### Second case: anomaly leading to the discovery of catalytic RNA

Until the very early 1980, it was unanimously believed that only proteins could act as biological catalysts (enzymes). However, unanticipated findings that emerged during ‘normal science’ experimental projects led to a recognition that some RNA species were also capable of conducting biological catalysis. The discovery of RNA enzymes (’ribozymes’) brought about a far-reaching conceptual change from protein-exclusive biological catalysis to catalysis by both proteins and RNA.

Catalytic RNA molecules were independently and concomitantly discovered in the Yale University laboratory of Sidney Altman and in the laboratory of Thomas Cech at the University of Colorado. The original objective of Cech’s ‘normal science’ project was to identify proteins that regulate the transcription of the nucleolar ribosomal RNA coding genes (rDNA) in the ciliated protozoan *Tetrahymena*. These genes were known beforehand to include a spliced intervening sequence. When Art Zaug and Cech followed transcription of the rDNA in crude extracts of *Tetrahymena* nuclei they discerned both mature rRNA transcripts and an excised fragment of the rDNA intervening sequence (Zaug & Cech, 1980). To isolate from the nuclear extract presumed protein factor(s) that excise the intervening sequence, Cech and associates used as substrate purified unprocessed rRNA. As anticipated, incubation of this rRNA with the extract resulted in appearance of the excised intervening sequence. However, an entirely unexpected result was that the same excised fragment appeared in control samples that contained purified rRNA but no extract. Initially thinking that this anomaly must have been a mistake, Cech told Zaug: “*Well Art, this looks very encouraging, except you must have made a mistake making up the control sample*” (Cech, 1990). Repeated experiments, however, yielded the same result, hinting that the primary rRNA transcript may act on itself to excise the intervening sequence and rejoin its two flanking segments. Much work had been subsequently invested in substantiation of this surprising observation and in the elucidation of the detailed mechanism of the self-catalyzed splicing of the rRNA [reviewed in (Cech, 1988, 1990)]. Thus, in this case too, a project that began as ‘normal science’ activity yielded unanticipated anomaly which inaugurated verified discovery that replaced the model of proteins as the only biological catalyzers by new understanding the biological catalysis is carried out by some moleculal species of both protein and RNA.

That RNA can act as catalyst was discovered in a concomitant independent study of a different system. This work of Altman and associates also began as a ‘normal science’ project that aimed at the elucidation of the mechanism of cleavage of pre-tRNA molecules by the ribonucleoprotein enzyme Ribonuclease P (RNase P). However, unexpected observations led to the discovery that the RNA subunit of RNase P acted alone to catalyze cleavage of the pre-tRNA whereas the protein subunit, which was devoid of catalytic activity, only accelerated the RNA-catalyzed reaction [(Guerrier-Takada et al., 1983); reviewed in (Altman, 1990, 2000)].

## Anomaly triggered discovery is but one of several paths to the discovery of factual biological entities

As was shown above, the discoveries of the factual biological entities—archaea, RNA splicing, and catalytic RNA conformed to the Kuhnian model discoveries triggered by observational or experimental anomalies (Kuhn, 1962a, 1962b). However, a closer look reveals that this model is by no means a fit-all universal path to discoveries in the life sciences and that alternative approaches also led to discoveries of new unpredicted factual biological entities. This concluding section offers a passing glance at historically proven other effective strategies for discovery of factual entities in biology.

### Discovery by deployment of specific instruments or techniques

Use of novel devices or experimental methodologies effectively opened ways to discoveries of new biological entities. For instance, deployment of microscopes of specific resolving power propelled discoveries of certain cell types, of subcellular structures, and of viruses, (Bonifacino, 2020; Hayat, 1987). In other cases use of the ultracentrifuge prompted the discoveries of mitochondria and microsomes (Claude, 1944) and of lysosomes (De Duve et al., 1953, 1955).

### Discovery by interrogation of large datasets

Another non-Kuhnian path to discovery employs computer-aided searches of large ‘Omics’ databases. In general, computer-aided queries of large databases endeavor to discover hitherto hidden entities or regularities (Leonelli, 2013; Philippi & Köhler, 2006). Just few of the myriad examples of efficacious deployment of this approach are the interrogation of the human genome for the discovery of multiple disease genes (Antonarakis, 2021) or of drugs that are targeted at specific sub-populations or persons (Bachtiar et al., 2019). Likewise, probing of the proteome database discovered diagnostically important cancer biomarkers, (Kang, 2021) and new targets for anti-cancer drugs (Kurimchak et al., 2020).

### Theory-free experimental discoveries

Many major discoveries were made by stepwise methodological investigations with no guiding theory and with only an open biological question [for recent analysis see (Fry, 2022)]. This general approach had been successfully deployed for the discoveries of the protein biosynthesis (‘translation’) apparatus, (Rheinberger, 2006) the cyclic AMP signaling molecule, (Sutherland, 1970, 1992) and the non-lysosomal ubiquitin–proteasome system of intracellular protein breakdown (Hershko, 2005).

### Potential future approach to discovery: employment of artificial intelligence

As discussed in Sect. 1, because scientific discoveries were traditionally viewed as reason-defying unique occurrences, discovery-generating algorithms were thought to be unattainable. However, very recent developments in computer sciences introduced a prospect of using generative artificial intelligence to create scientific hypotheses and accelerate and even produce new discoveries. Although substantial hurdles, such as poor datasets quality and stewardship, stand on the way of making this possibility a reality, deployment AI methods to generate new scientific discoveries is currently holding enticing potential (Wang et al., 2023).

## Data Availability

Not applicable.

## Footnotes

1 Scientific discovery is an important, but not an only or necessarily obligatory component of the multifarious processes of acquisition of new knowledge about the natural world. Addressing the multitude of philosophical questions and ideas on the scientific pursuit of knowledge about nature is beyond the scope of this paper and the relevant philosophical literature is much too vast to be adequately addressed here. This paper is limited to historical and philosophical thinking on discovery at large and particularly on discovery of factual biological entities. Cited publications on these topics were selected from a more expansive body of work.

2 This paper uses lexical definitions of the term ‘discovery’: “[an] act of finding or learning something for the first time” (Britannica Dictionary) and “[a] process of learning something the was not known before or finding (...) something that was not known about before” (McMillan Dictionary).

3 Disavowing the narrow ‘eureka moment’ view of discovery, Carl Kordig wrote: “Real discoveries are more than initially plausible [hypothesis]. They are well established and justified. Only what is justified is a discovery” [(Kordig, 1978) p. 112]. According to Norwood Hanson, a discovery is certified as such only after it is crystallized into ‘finished research report’ [(Lund, 2010) p. 26]. Extrapolating from the discovery of bacteriophages, William Summers generalized on the process that follows discovery of a “fact”: “…during (that) integration process, the fact seems to expand into a stable discovery by accretion of other facts, contexts, theoretical explanations, and general understanding. Thus, discovery seems to be a process rather than event” (Summers, 2021).

4 It may be argued that since theories, unlike physical objects or systems, do not exist in the real world, their birth is a process of (mental) ‘generation’ or ‘creation’ rather than ‘discovery’. This view notwithstanding, philosophers of science routinely use the term ‘discovery’ to describe birth of theories.

5 First introduced by Charles Peirce as a new type of logical reasoning distinct from inductive or deductive logic, abductive logic infers from extant data and especially from unexpected irregular phenomena (Fann, 1970; Kraus, 2003).

6 This so-called PaJaMa experiment, named after the last names of its three authors: Pardee, Jacob, and Monod, was described in detail in the original paper (Pardee et al., 1959) and in many subsequent articles [i.e., (Schaffner, 1974; Pardee, 2002, 2003); (Fry, 2016a) pp. 371-381]

7 Schaffner limited the context of justification in this case to circumstantial supporting evidence derived from the PaJaMa experiment itself or from inferences thereof. Under a more comprehensive view, justification (corroboration) of the repressor theory was accomplished only after the repressor was physically isolated and its specific binding to both the target regulatory DNA segment and to the inducer molecule were experimentally demonstrated (Gilbert & Müller-Hill, 1966, 1967).

8 Such strategy which ideally generates a plausible theory, differs from algorithm that produces guaranteed correct theory.

9 Representative cases of this category were the discoveries of radio waves, the neutrino, and elements predicted to fill empty spots in the periodic table (Kuhn, 1962a).

10 Representative cases of this category were the discoveries of oxygen, the planet Uranus, and X-rays (Kuhn, 1962a).

11 In an 1854 lecture at the University of Lille, Louis Pasteur famously underscored the import of the skill and talent of the scientist in exploiting an opportune observation: “In the fields of observation, chance favours only the prepared mind”.

12 Woese’s scientific achievements were amply recognized in his lifetime: MacArthur Fellowship (1984); elected member of the National Academy of Sciences USA (1988) and of the Royal Society (2006); the Dutch Royal Academy of Science’s Leeuwenhoek Medal (1992); the US National Medal of Science (2000); and the Crafoord Prize in Biosciences (2003). For biographical details and appreciation of Woese’s scientific contributions see (Sapp, 2008; Nair, 2012; Sapp & Fox, 2013; Goldenfeld, 2014; Gold, 2014; Koonin, 2014; Moore, 2014; Luehrsen, 2014; Quammen, 2018).

13 For a history of the evolution of Chatton’s classification and terminology see (Katscher, 2004).

14 This naming reflects the 18th century perception of a chaotic universe of microscopic organisms.

15 The belief that the bacterial world comprised of multitude of fixed species, (monomorphism) was thrown into turmoil in the second half of the 19th century. An alternative view (polymorphism) claimed that there is only a single or very few bacterial species and that their different shapes and even transformation into yeast and fungi are consequences of changing conditions. Robert Koch ultimately settled the dispute between monomorphism and polymorphism on the side of the former. He showed in classical experiments that isolated single types of bacteria retained their morphologies and properties over many generations [for a history of the debate and Koch’s experiments see (Fry, 2020)].

16 “Any good biologist finds it intellectually distressing to devote his life to the study of a group that cannot be readily and satisfactorily defined in biological terms; and the abiding intellectual scandal of bacteriology has been a clear concept of a bacterium.” [(Stanier & Niel, 1962) p. 2].

17 "An eminent contemporary bacteriologist, van Niel, who is noted for his taxonomic studies on several groups of bacteria, has expressed the opinion that it is a waste of time to attempt a natural system of classification for bacteria, and that bacteriologists should concentrate instead on the more humble practical task of devising determinative keys." [(Stanier et al., 1957) p. 296].

18 Beginning in the early 1950s, paleobiologists identified Precambrian fossils of >3 billion years old bacteria [evidence that was already available at the onset of Woese’s work was summarized by (Schopf, 1975)].

19 Globins are proteins that form hemoglobins together with a non-proteinaceous heme molecule. Starting by comparison of patterns of tryptic oligopeptides of homologous globins from different species, (Zuckerkandl et al., 1960) Zuckerkandl later progressed to finer comparisons of their amino acid sequences (Zuckerkandl & Schroeder, 1961; Zuckerkandl & Pauling, 1962; Pauling & Zuckerkandl, 1963).

20 About seven years before Zuckerkandl and Pauling introduced their hypothesis, Francis Crick raised an abstract idea that had no experimental support, of applying molecular evolutionary clock to build taxonomies: “Biologists should realise that before long we shall have a subject which might be called ‘protein taxonomy’- the study of the amino acid sequences of the proteins of an organism and the comparison of them between species. It can be argued that these sequences are the most delicate expression possible of the phenotype of an organism and that vast amounts of evolutionary information may be hidden away within them.” [(Crick, 1958) p. 142].

21 Facsimiles of the Crick-Woese letters (1962-1973) are in the Wellcome Collection Web site: https://wellcomecollection.org/works/ej5p7b5z/items?canvas=15

22 Facsimile of this June 24, 1969, letter is in the Wellcome Collection: https://wellcomecollection.org/works/ej5p7b5z/items?canvas=40

23 The molecular biologist Sol Spiegelman, Woese’s colleague in the Department of Microbiology, University of Illinois at Urbana-Champaign was recruited in 1969 by Columbia University. As Woese mentioned in his letter to Crick, upon departing for New York Spiegelman gave Woese his sequencing equipment. Woese’s acceptance of this apparatus suggests that by that time he had already made up his mind to apply Sanger’s methodology for his bacterial taxonomy project.

24 Crick and Sanger were colleagues in the Cambridge MRC Unit.

25 This premise was supported by prior results of RNA-DNA hybridization experiments that indicated that despite their conservation, (Yankofsky & Spiegelman, 1963) rRNA molecules from different organisms did diverge as reflected by the lower stability of heterologous (inter-species) ribosomal RNA-DNA hybrids relative to homologous (intra-species) hybrids (Moore & McCarthy, 1967; Bendich & McCarthy, 1970; Pace & Campbell, 1971).

26 Discovered in 1964, (Rosset et al., 1964) 5S RNA, (‘S’ stands for Svedberg unit - a measure of the rate of sedimentation of a macromolecule under high centrifugal acceleration), was later found to be a part of the large subunit of every bacterial, archaeal, and eukaryotic ribosome. Full sequences of 5S RNA molecules from two bacterial species were determined prior to Woese’s work (Brownlee et al., 1967; DuBuy & Weissman, 1971).

27 Bacterial ribosomes (70S) comprise of two nucleoprotein subunits of 50S and 30S. The RNA components of these subunits are 23S + 5S for the 50S particle and 16S for the 30S subunit. Larger ribosomes (80S) of eukaryotic cells comprise of 60S and 40S nucleoprotein subunits whose respective RNA components are 28S + 5.8S + 5S and 18S.

28 Members of the Woese laboratory in the 1970s and early 1980s included Linda Bonen, George Fox, Jane Gibson, Bobby Lewis, Kenneth Luehrsen, Linda Magrum, Jack Maniloff, Gary Olsen, Ross Overbeek, Kenneth Pachman, Henry Schaup, Mitchell Sogin, David Stahl, Lorraine Sutton, Tsuneko Uchida, and Lawrence Zablen.

29 Degree of genetic relatedness of two compared species was determined under the assumption that sequence identity between oligomers of 6 nucleotides or more is highly indicative of their common ancestry. Extents of genetic closeness of paired bacterial species were expressed by their association coefficient, Sab, defined as the fraction of the total number of nucleotides in any pair of catalogues A and B of oligomers of 6 or more nucleotides that are found in sequences common to the two catalogues [(Woese, 1987) p. 228].

30 Three to four weeks were needed to complete a catalogue of 16S rRNA oligomer sequences from one bacterial species (Sapp & Fox, 2013).

31 Primary pattern is that of the 2D electrophoretically separated oligonucleotides products of T1 RNase digestion of 16S rRNA. “Secondary” is the 1D electrophoretic resolution of products of digestion of the primary oligomers by ribonucleases other than T1 (see Fig. 1A).

32 Following the introduction of the Sanger DNA sequencing technique, (Sanger et al., 1977) the number of sequenced 16S rRNA genes of different species and their deduced rRNA transcripts, grew at accelerating pace such that by 1994 sequences of 16S rRNAs were determined for more than 1500 prokaryotes (Olsen et al., 1994).

33 For lists of the analyzed bacterial families and their places in phylogenetic trees see (Fox et al., 1980; Woese, 1987; Olsen et al., 1994).

34 These trees represented relative evolutionary distances between species and phyla with no determinate time scale.

35 Capacious whole genome data that were gathered in the recent two decades bred alternative bacterial classification systems. Yet, no consensus had been reached yet on taxonomic and nomenclatural standards for bacteria (Hugenholtz et al., 2021).

36 As originally proposed, (Woese & Fox 1977a) the hypothetical progenote ancestor of all forms of life existed before genotype and phenotype became firmly coupled [for the presumed characteristics of the progenote and its relationship to the putative Last Universal Common Ancestor (LUCA) see (Gogarten, 2019)].

37 Very recent experimental results pointed to members of the Asgard archaeal phylum as likely ancestors of eukaryotes, (2.2.6 below). However, these ancient cells are positioned at the archaea/eukaryotes branching point, downstream from the hypothetical progenote ancestor.

38 Because of their larger size and high content of modified nucleotides, 18S rRNA chains of the smaller eukaryotic ribosomal subunit were harder to sequence then their analogous prokaryotic 16S rRNA chains.

39 Fox and Balch have known one another since 1973 when they attended together a course in microbial ecology at the Woods Hole Marine Laboratory.

40 Fox’s skepticism was due to the trivial possibility that because of mix-up, the source of the examined 16S rRNA was not the methanogenic microorganism but some other organism(s) (eukaryotic? eukaryotic/prokaryotic mixture?). This alternative explanation was excluded after rRNA chains from the original methanogen and from a different methanogenic species yielded again exceptional non-prokaryotic and non-eukaryotic fingerprint patterns and nucleotide sequences.

41 This assertion was hinted by earlier unpublished finding in the Woese laboratory that the sequence of methanogen 5S RNA was more similar to that of eukaryotes than to prokaryotes (Sapp & Fox, 2013)

42 As noted, (Woese & Fox, 1977b) the similarity between chloroplast and bacterial rRNA sequences provided supporting molecular evidence to the hypothesis that the origin of chloroplasts were protobacteria which after engulfment by proto-eukaryotic cells, became stable endosymbionts.

43 First termed ‘Primary Kingdoms’ or ‘Ur kingdoms’, (Woese & Fox, 1977b) these three lines of decent were later renamed ‘Domains’ (Woese et al., 1990).

44 Eubacteria and archaebacteria were both defined as prokaryotes based on their unicellularity, absence of membrane-enveloped nucleus, and lack of intracellular organelles.

45 Prior to the negative reception of the published paper, (2.2.4 below) it encountered resistance already upon submission to the Proceedings. One reviewer, criticizing the methodology of the work and rebuffing the claimed three-domain phylogenetic tree, recommended that the paper be rejected (Pace et al., 2012).

46 Lyons, Richard D. New York Times, Vol. CXXVII, No. 43748, November 3, 1977; https://timesmachine.nytimes.com/timesmachine/1977/11/03/issue.html. The Times was informed about the discovery by NASA, which funded in part Woese’s research work.

47 “Woese's solitary years at his light table [used to visualize autoradiograms] had left him with a reputation as an odd person, “a crank, who was using a crazy technique to answer an impossible question” as one researcher put it. “His tiny snippets of rRNAs were considered too fragmentary to be reliable indicators of evolutionary relationships” said [Norman] Pace. The Yale University molecular biologist Alan Weiner recalled that many leading biologists thought Woese was “crazy,” and that his RNA tools couldn't possibly answer the question he was asking.” (Morell, 1997).

48 Parallel to this main objective, Woese also continued his original bacterial taxonomy project, (Woese et al., 1976; Fox et al., 1977b; Fox et al., 1980; Woese et al., 1980b; Woese et al., 1985; Woese, 1987) and studied secondary structure of 16S rRNA (Woese et al., 1983; Gutell et al., 1985)

49 Prominent among those were the University of Munich laboratory of Otto Kendler, (archaeal cell wall chemistry) Wolfram Zillig’s laboratory at the Munich Max-Planck Institute, (archaeal DNA dependent RNA polymerase) and the South Dakota University laboratory of Thomas Tornabene, (archaeal membrane lipids).

50 Found in nature in salt-saturated brine, halophilic archaea, (haloarchaea), require high concentration of NaCl (>2 M) for their survival and growth. Thermoacidophile archaea grow in acidic environment (pH 2-4) at high temperatures (80 to >100 °C).

51 Early identified archaea were all extremophiles that grew in harsh habitats such as high-salt brines, hot geothermal environments, or strictly anoxic milieu in which even a trace of oxygen is lethal. Because such environments were conceived to mirror conditions at the beginning of life on earth, archaea were seen as the likely earliest living forms that preceded bacteria in evolution. However, later discoveries of mesophilic archaea that grow under temperate conditions, (DeLong, 1992; Fuhrman et al., 1992; DeLong, 1998) suggested that despite their referential name, archaea were not older than eubacteria (Eme & Doolittle, 2015).

52 Although these early studies identified in the examined archaeal genome higher proportions of bacteria-like than eukaryote-like genes, later discovered archaeal phyla were found to be more closely related to eukarya (see 2.2.6).

53 Yet, prominent traditional evolutionists such as Ernst Mayr, (Mayr, 1990, 1998) and Lynn Margulis, (Margulis, 1996) insisted that bacteria and archaea belonged to a single prokaryote domain. Woese countered that Mayr’s taxonomy was focused on the relatively young eukaryotic branch of the phylogenetic tree. By contrast, bacteria and archaea had much longer evolutionary histories that engendered their significantly higher diversity (Woese, 1998a). Mounting evidence eventually corroborated the division between eubacteria and archaea and the three-domain model of the phylogenetic tree prevailed.

54 Discussion of this remarkable finding is beyond the scope of the present paper. Some recent developments in the rapidly moving study of Asgard archaea can be found in (Zaremba-Niedzwiedzka et al., 2017; Cunha et al., 2018; Cunha et al., 2022; Hatano et al., 2022; Rodrigues-Oliveira et al., 2023; Wu et al., 2022).

55 Sharp and his coworkers Susan Berget and Claire Moore, were at the MIT. Roberts and his associates Louise Chow, Richard Gelinas, and Tom Broker were working at the Cold Spring Harbor Laboratory.
